# Yield of Long-Read Genome Sequencing for Rare Disease Diagnosis in Short-Read Genome Negative Cases

**DOI:** 10.64898/2026.09.09.26362331

**Published:** 2026-09-10

**Authors:** Georgia Pitsava, Krista Bluske, Rebekah Barrick, Ivan De Dios, Cathy Duong, Kirsten Blanco, Sami Belhadj, Nida Karra, Sarah L. Stenton, Arthur Garcia, Erica Smith, Miguel Almalvez, Arthur Ko, John Harting, Stuti Joshi, Kenny Chen, Charles Hadley King, Jonathan LoTempio, Jessica Albert, Kinga M. Bujakowska, Leandros Boukas, UCI-GREGoR Consortium, Rachid Karam, Seth I. Berger, Emmanuèle C. Délot, Changrui Xiao, Eric Vilain

## Abstract

**Background:**

A growing body of work has highlighted advantages of long-read genome sequencing (LR-GS) over short-read genome sequencing (SR-GS) with regards to the detection and interpretation of pathogenic variants. However, the incremental diagnostic yield of LR-GS over SR-GS for patients with suspected Mendelian conditions has not been systematically characterized.

**Methods:**

We performed LR-GS on 144 cases that previously had undergone SR-GS through the Pediatric Mendelian Genomics Research Center at the University of California, Irvine (PMGRC-UCI, a founding center of the GREGoR Consortium), of which 107 remained undiagnosed after SR-GS.

**Results:**

We identified new molecular diagnoses in 13 cases, five of which could not have been detected with SR-GS, corresponding to an incremental diagnostic yield of 4.7%. These five diagnoses leveraged distinct capabilities of LR-GS: the detection of variants in short-read “dark” regions, the detection of structural variants and tandem repeat expansions, and the identification of *de novo* variants using only one biological parent. In two of these five cases, confirmation of the diagnosis also relied critically on identifying aberrant transcript production via RNA-seq, while in one other case LR-GS-based CpG methylation profiling further supported the diagnosis through detection of abnormal methylation at episignature-associated CpGs.

**Conclusions:**

In this paired comparison of LR-GS and SR-GS, LR-GS provided an incremental diagnostic yield of 4.7% among cases that remained undiagnosed after SR-GS. Together, our findings demonstrate that LR-GS can resolve a meaningful subset of cases beyond the reach of SR-GS while simultaneously expanding the range of genomic and epigenomic mechanisms accessible to a single sequencing assay.

## Full Text Availability

The license terms selected by the author(s) for this preprint version do not permit archiving in PMC. The full text is available from the preprint server.

